# A Retrospective Analysis of Extended-Spectrum β-Lactamase (ESBL)-Producing Enterobacterales Bacteremia in Tertiary Care Hospitals in the United Arab Emirates

**DOI:** 10.7759/cureus.99381

**Published:** 2025-12-16

**Authors:** Ali Al Hassani, Aqeel Saleem, Zaid Al Hassani, Muhammed Rashid, Aya Shubbar, Mohamed Abdilsalhen, Aisha Al Naqbi, Latifa Al Kamali, Lujein Watad, Merna Abdelsalhen, Mustafa Al Hassani, Ahmed Al Hammadi

**Affiliations:** 1 Infectious Disease, Tawam Hospital, Al Ain, ARE; 2 Infectious Disease, Sheikh Tahnoon Medical City, Al Ain, ARE; 3 General Medicine, Sheikh Tahnoon Medical City, Al Ain, ARE; 4 Internal Medicine, Sheikh Tahnoon Medical City, Al Ain, ARE; 5 Infectious Disease, Al Rahba Hospital, Al Ain, ARE

**Keywords:** antimicrobial resistance, antimicrobial stewardship, bloodstream infection, carbapenems, empirical therapy, esbl-producing enterobacterales, extended-spectrum β-lactamase, mortality, piperacillin–tazobactam, risk factors

## Abstract

Introduction

The global increase in extended-spectrum β-lactamase (ESBL)-producing *Enterobacterales *(ESBL-E) has significantly complicated the treatment of bloodstream infections (BSIs). This study aimed to assess the epidemiology, antimicrobial resistance patterns, sources of infection (including vascular catheter-related and organ-based infections progressing to sepsis), and clinical outcomes of ESBL-associated BSIs in patients treated at two tertiary care hospitals in the United Arab Emirates (UAE), with particular focus on the impact of empirical antibiotic selection.

Methods

We conducted a retrospective cohort study of 176 patients with *Enterobacterales *BSIs screened at Tawam Hospital and Sheikh Tahnoon Medical City (STMC) in the UAE between January 1, 2024, and January 1, 2025. All consecutive adult patients (≥18 years) with laboratory-confirmed monomicrobial *Escherichia coli (E. coli) *or *Klebsiella pneumoniae (K. pneumoniae) *bacteremia and complete clinical and microbiological data who met eligibility criteria were included in the final cohort (n=172). Exclusion criteria included patients with polymicrobial bacteremia, isolates from non-blood sources, pediatric patients (<18 years), incomplete records, or carbapenem-resistant isolates. Data collected included patient demographics, microbiological findings, antimicrobial regimens, and clinical outcomes.

Results

Of the 176 patients screened, 172 met the inclusion criteria (four patients with carbapenem-resistant isolates were excluded), and 76 (44.2%) had ESBL-producing isolates; *E. coli *(44 cases, 58.4%) and *K. pneumoniae* (32 cases, 42.6%) predominated. ESBL strains were highly resistant to ceftriaxone (86.8%) and ciprofloxacin (71.4%) yet completely susceptible to carbapenems. Empirical carbapenem therapy was associated with numerically lower 30-day mortality compared with piperacillin-tazobactam (14.3% vs. 31.3%, p=0.12), although this difference did not reach statistical significance. Delayed appropriate therapy (>48 hours) increased mortality risk (odds ratio (OR): 2.9, 95% confidence interval (CI): 1.5-5.6, indicating nearly threefold higher odds of death); predictors of ESBL production were prior cephalosporin use (OR: 3.1, p<0.001), ICU admission (OR: 2.7, p=0.002), recent hospitalization (OR: 2.3, p=0.001), and Charlson Comorbidity Index (CCI) >4 (OR: 1.8, p=0.05).

Conclusions

Nearly half of carbapenem-susceptible *E. coli* and *Klebsiella* BSIs in the two centers in the UAE were ESBL-producing. Carbapenems retained full activity and offered a clinically meaningful survival advantage in high-risk patients. These findings support early risk-based carbapenem use and highlight the importance of antimicrobial stewardship and rapid diagnostics.

## Introduction

Extended-spectrum β-lactamase (ESBL)-producing *Enterobacterales* have emerged as a major challenge in clinical infectious diseases. Since their first description in the 1980s, these organisms have become increasingly prevalent worldwide, with reported rates exceeding 50% in some regions [1]. Bloodstream infections (BSIs) caused by ESBL-producing bacteria are particularly concerning, as they are associated with significantly higher mortality compared to non-ESBL infections [1,2]. The United States Centers for Disease Control and Prevention (CDC) has identified 18 bacterial pathogens as urgent, serious, or concerning threats, including carbapenem-resistant *Enterobacterales* (CRE) and ESBL-producing *Enterobacterales* (ESBL-E) [3]. Recent surveillance data show an increase of more than 50% in both ESBL-E and CRE cases between 2019 and 2020 [4], and a 50% rise in hospital and community-acquired infections caused by ESBL-producing Gram-negative bacilli between 2012 and 2017 [3]. However, published data on ESBL BSIs from the United Arab Emirates (UAE) and the wider Gulf region remain limited, and local epidemiology is not well characterized.

Management of ESBL BSIs remains challenging. Conventional susceptibility testing may not detect ESBL production promptly, delaying appropriate therapy. The optimal empirical antibiotic regimen is still debated, with ongoing discussions on the role of carbapenems versus alternative agents such as piperacillin-tazobactam [2,5]. In addition, the global rise of carbapenem-resistant organisms necessitates careful stewardship of these critical antibiotics. This study aimed to comprehensively evaluate the epidemiology, resistance patterns, and clinical outcomes of ESBL BSIs in two tertiary care hospitals in the UAE (Tawam Hospital and Sheikh Tahnoon Medical City (STMC), with particular attention to the impact of empirical antibiotic selection on mortality. Given the high prevalence of ESBL-E​​​, the ongoing debate regarding carbapenems versus non-carbapenem β-lactams, and the paucity of outcome data from the UAE, we focused on empirical therapy as a key modifiable factor that may inform local treatment strategies.

## Materials and methods

Study design and population

This retrospective cohort study was conducted at Tawam Hospital and STMC, two tertiary care centers in the UAE, between January 1, 2024, and January 1, 2025. During the study period, 176 adult patients with laboratory-confirmed monomicrobial *E. coli *or *K. pneumoniae* bacteremia were identified; after applying the eligibility criteria, 172 patients constituted the final study population.

Inclusion and Exclusion Criteria

Inclusion criteria were adults (≥18 years) with laboratory-confirmed monomicrobial *E. coli* or *K. pneumoniae* bacteremia and complete medical records containing microbiological data, treatment details, and clinical outcomes. Exclusion criteria included polymicrobial bacteremia in which ESBL-producing organisms were not the dominant pathogen, pediatric patients (<18 years), incomplete records, isolates obtained from non-blood sources, and carbapenem-resistant isolates (resistant to ertapenem or meropenem). Because patients with incomplete core microbiological, treatment, or outcome data were excluded, missing data for the primary analyses were minimal, and no imputation procedures were used.

Sampling and Sample Size

We used consecutive sampling and included all eligible patients during the study period; as this was a retrospective cohort, no a priori sample size calculation was performed.

Data collection

Data were extracted from electronic medical records and microbiology laboratory databases using a standardized case report form. The study population’s baseline demographics and comorbidities were captured a priori, including age, sex, hospital location, and comorbidity burden using the Charlson Comorbidity Index (CCI). Recent healthcare exposures within 90 days before bacteremia were recorded, including hospitalization, admission to long-term care facilities, invasive procedures such as surgery or catheterization, and prior antibiotic use (cephalosporins, fluoroquinolones, carbapenems, or piperacillin-tazobactam).

Microbiological data included organism identification (*E. coli* or *K. pneumoniae*) and antimicrobial susceptibility testing (AST) results for β-lactams (ceftriaxone, cefepime, piperacillin-tazobactam), carbapenems (meropenem, ertapenem), fluoroquinolones (ciprofloxacin), and trimethoprim-sulfamethoxazole. Identification and routine susceptibility testing were performed in the hospital microbiology laboratories according to Clinical and Laboratory Standards Institute (CLSI) methodology. ESBL production was confirmed using the phenotypic combined-disk method in accordance with CLSI recommendations [6].

Clinical characteristics included the source of bacteremia, classified as urinary tract infection (UTI), pneumonia, intra-abdominal infection, central line-associated bloodstream infection (CLABSI), skin and soft tissue infection (SSTI), or bone and joint infection (BJI). Severity of illness was assessed using the Pitt Bacteremia Score (0-1, 2-5, or >4). The need for ICU admission and mechanical ventilation at the time of bacteremia was documented. Mode of acquisition was defined as community-acquired if symptom onset occurred less than 48 hours after admission, or hospital-acquired if onset occurred 48 hours or more after admission.

Treatment data

Treatment data included the empirical antibiotic regimen initiated before susceptibility results were available, definitive antibiotic therapy adjusted according to susceptibility testing, and the timing of appropriate therapy. Appropriate empirical therapy was defined as administration of at least one antibiotic active against the isolate within 24 hours of blood culture collection. Delayed appropriate therapy was defined as administration of an effective antibiotic more than 48 hours after collection. The total duration of intravenous and oral antibiotic therapy was recorded. Appropriateness of the empirical regimen was determined retrospectively by comparing the initial treatment to the in vitro susceptibility profile of the bloodstream isolate once AST results were available.

Clinical outcomes

Clinical outcomes included 30-day and 90-day all-cause mortality, hospital length of stay (LOS), and duration of bacteremia, defined as the time between the first positive and the first negative blood culture. Repeat blood cultures were obtained at the discretion of the treating clinicians as part of routine care rather than by a study-specific protocol; duration of bacteremia was calculated only for patients with documented clearance cultures.

Definitions

ESBL production was confirmed using the phenotypic combined-disk method as recommended by the CLSI guidelines [6]. Appropriate empirical therapy was defined as administration of at least one antibiotic within 24 hours of blood culture collection that demonstrated in vitro susceptibility against the isolated pathogen. Delayed appropriate therapy was defined as the administration of an effective antibiotic more than 48 hours after blood culture collection. The source of bacteremia was determined according to the CDC and Prevention/National Healthcare Safety Network (CDC/NHSN) surveillance definitions, which outline criteria for classifying primary and secondary bloodstream infections [7].

Statistical analysis

Categorical variables were summarized as frequencies and percentages and compared using the chi-square (χ²) test when all expected cell counts were ≥5 and Fisher’s exact test when any expected cell count was <5. Continuous variables were assessed for normality using the Shapiro-Wilk test and reported as mean ± standard deviation (SD) for normally distributed data or as median and interquartile range (IQR) for non-normally distributed data. Parametric comparisons were performed with Student’s t-test, and non-parametric comparisons with the Mann-Whitney U test. Multivariable logistic regression models were used to identify independent risk factors for (i) ESBL production and (ii) 30-day and 90-day mortality. Models were adjusted for potential confounders, including age, CCI, Pitt Bacteremia Score, ICU admission, source of infection, and appropriateness of empirical antibiotic therapy.

Kaplan-Meier survival curves were generated to compare 30-day and 90-day survival between ESBL-positive and ESBL-negative bacteremia, and between appropriate and inappropriate empirical therapy, and the corresponding log-rank p-values are reported in the Results section. Survival differences were assessed using the log-rank test. All analyses were conducted using SPSS Statistics version 27.0 (IBM Corp., Armonk, NY), and a two-tailed p-value of less than 0.05 was considered statistically significant.

Ethical considerations

The study was approved by the Tawam Human Research Ethics Committee (Approval No.: MF2058-2025-1232). The requirement for informed consent was waived due to the retrospective design. All patient data were anonymized to ensure confidentiality.

## Results

Four patients with carbapenem-resistant isolates were excluded from the initial cohort of 176. A total of 172 patients with *Enterobacterales* bacteremia were included in the final analysis, of whom 76 (44.2%) had ESBL-producing isolates. The median age was 72 years (IQR: 58-84) with no significant difference between ESBL-positive and ESBL-negative groups. Males predominated in both groups (ESBL+ 44 cases (58.5%) vs. ESBL− 52 cases (54.5%), p=0.42). The most frequent comorbidities were diabetes mellitus (73 cases, 42.3%), chronic kidney disease (55 cases, 31.8%), and malignancy (49 cases, 28.4%).

**Figure 1:**
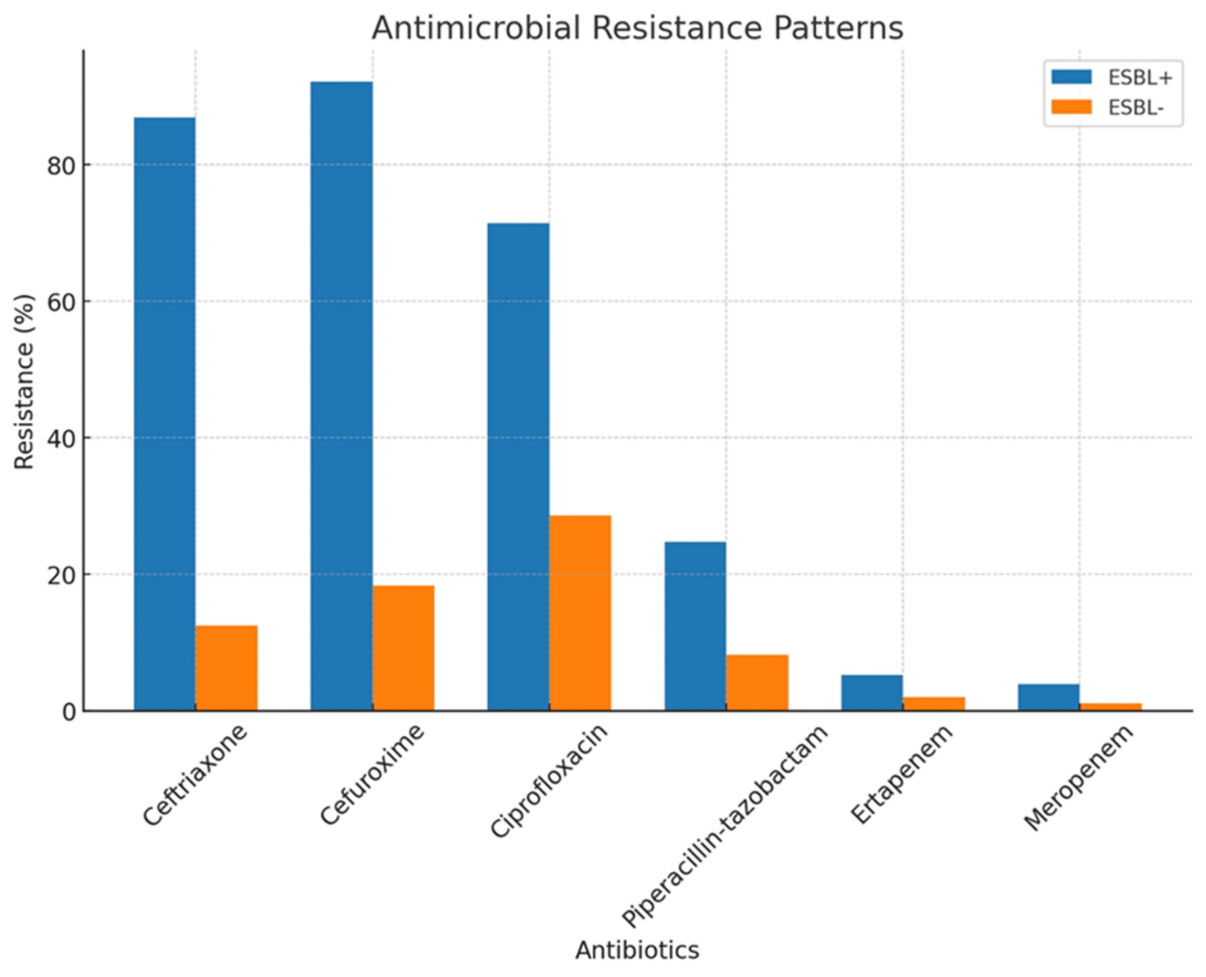
Antimicrobial resistance patterns in ESBL-positive and ESBL-negative isolates Percentage resistance among ESBL-producing (blue) and non-ESBL (orange) isolates. ESBL-positive isolates had higher resistance to ceftriaxone (86.8% vs. 12.5%; p<0.001), ciprofloxacin (71.4% vs. 28.6%; p<0.001), and piperacillin-tazobactam (25.0% vs. 8.3%; p=0.003). Carbapenem activity was preserved in both groups: ertapenem (0.0% vs. 2.0%; p=0.50) and meropenem (0.0% vs. 1.0%; p=1.00). Statistical tests: chi-square (χ²); Fisher’s exact when any expected cell <5. Units: y-axis = % resistant ESBL: extended-spectrum β-lactamase

Among ESBL-positive isolates, *E. coli* (44 cases, 58.4%) and *K. pneumoniae* (32 cases, 42.6%) accounted for the isolates. ESBL-positive strains demonstrated significantly higher resistance to third-generation cephalosporins (ceftriaxone: 86.8% vs. 12.5%, p<0.001) and ciprofloxacin (71.4% vs. 28.6%, p<0.001), consistent with the typical ESBL phenotype (Table 1, Figure 1). Resistance to piperacillin-tazobactam was approximately three times higher in ESBL-positive isolates (25.0% vs. 8.3%, p=0.003), underscoring the need for caution when using this agent empirically in suspected ESBL infections. In contrast, all ESBL-positive isolates were susceptible to carbapenems (ertapenem: 100%; meropenem: 100%), with no statistically significant difference in resistance compared to ESBL-negative isolates (p>0.15). These findings highlight the limited role of non-carbapenem β-lactams in ESBL bacteremia and support the use of carbapenems as the cornerstone of definitive therapy.

**Table 1:** Antimicrobial resistance patterns in ESBL-positive and ESBL-negative isolates Comparative resistance rates for ESBL-producing (ESBL+) and non-ESBL (ESBL-) isolates. ESBL-positive isolates had higher resistance to ceftriaxone (86.8% vs. 12.5%; p<0.001), ciprofloxacin (71.4% vs. 28.6%; p<0.001), and piperacillin–tazobactam (25.0% vs. 8.3%; p=0.003). Carbapenem activity was preserved in both groups: ertapenem (0.0% vs. 2.0%; p=0.50) and meropenem (0.0% vs. 1.0%; p=1.00). Statistical tests: chi-square (χ²) when all expected cell counts were ≥5 and Fisher’s exact test when any expected cell count was <5 or the sample size was small ESBL: extended-spectrum β-lactamase

Antibiotic	ESBL+ (n=76)	ESBL- (n=96)	P-value
Ceftriaxone	86.80%	12.50%	<0.001
Ciprofloxacin	71.40%	28.60%	<0.001
Piperacillin-tazobactam	25.00%	8.30%	0.003
Ertapenem	0.00%	2.00%	0.5
Meropenem	0.00%	1.00%	1

**Figure 2:**
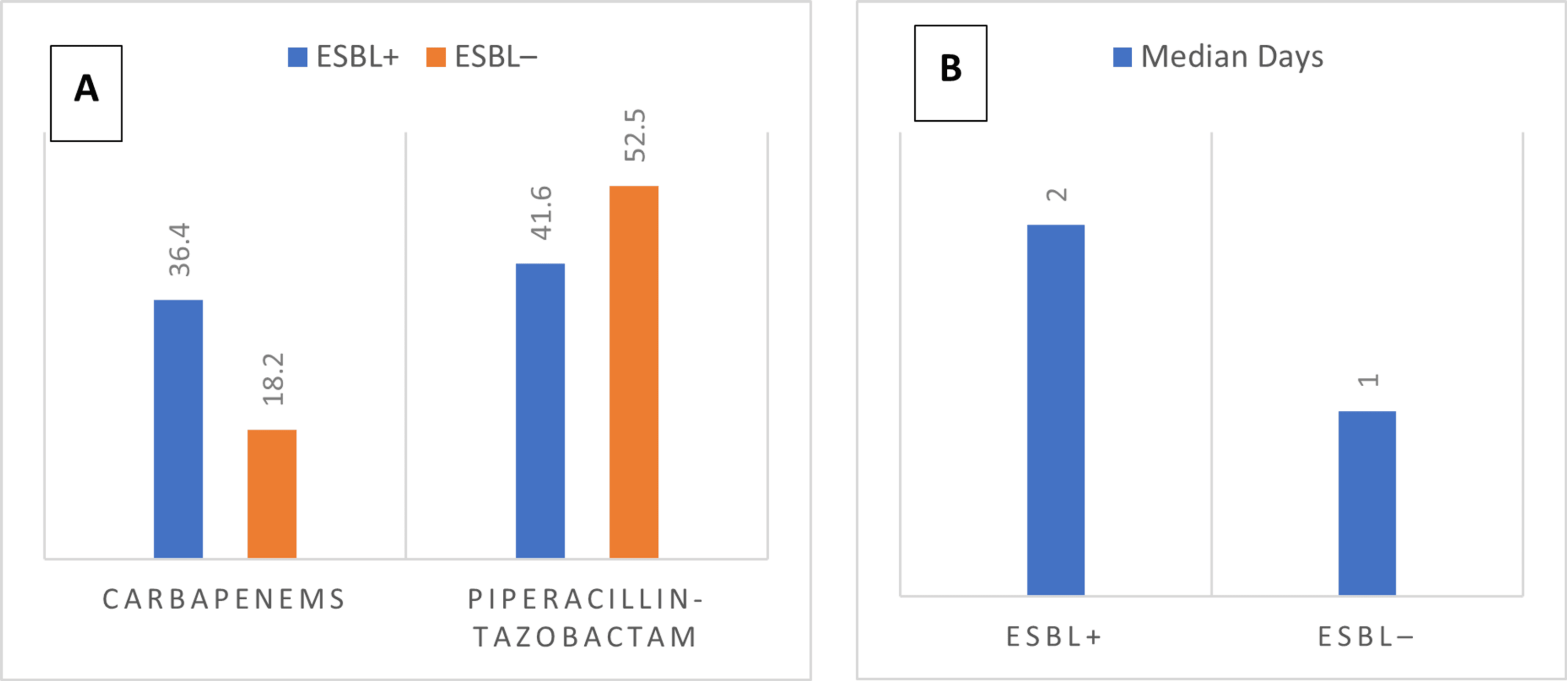
Empirical therapy and time to appropriate treatment in ESBL bacteremia (A) Empirical antibiotic regimens differed significantly between groups, with carbapenems prescribed more often in ESBL-positive cases (36.4% vs. 18.2%, p=0.008). Piperacillin–tazobactam remained the most common empirical agent overall. Statistical test (A): chi-square (χ²)/Fisher’s exact as appropriate. (B) Median time to appropriate therapy was significantly longer in ESBL-positive cases (2 days vs. 1 day, p = 0.008). Statistical test (B): Mann–Whitney U. Units: days ESBL: extended-spectrum β-lactamase

Empirical regimens differed significantly between ESBL-positive and ESBL-negative cases. Carbapenems were prescribed more often in ESBL-positive patients (28 cases, 36.4%) vs. 17 cases (18.2%), p=0.008), while piperacillin-tazobactam was the most common empirical agent overall (41.6% vs. 52.5%). Median time to appropriate therapy was longer in ESBL-positive cases (two days vs. one day, p=0.008), indicating delays in starting active treatment for resistant infections (Figures 2A, 2B).

ESBL-producing bacteremia was associated with worse outcomes than non-ESBL cases (Table 2). Thirty-day mortality was higher in ESBL-positive patients (18 cases (23.7%) vs. 12 cases (12.5%), p=0.06), and this difference was significant at 90 days (26 cases (34.2%) vs. 17 cases (17.7%), p=0.02). Multivariable logistic regression analysis confirmed ESBL production as an independent predictor of mortality (odds ratio (OR): 2.1, 95% confidence interval (CI): 1.2-3.8).

**Table 2:** Clinical outcomes in ESBL-positive and ESBL-negative bacteremia Summary of clinical outcomes comparing ESBL-positive and ESBL-negative bacteremia. ESBL-positive cases had higher 90-day mortality (34.2% vs. 17.7%; p=0.02) and longer hospital LOS (median 15 vs. 10 days; p=0.004). Thirty-day mortality (23.7% vs. 12.5%; p=0.06) and ICU admission (44.7% vs. 32.3%; p=0.10) did not reach significance. Statistical tests: proportions by chi-square (χ²)/Fisher’s exact; LOS by Mann–Whitney U. Units: mortality and ICU as % of patients; LOS in days ESBL: extended-spectrum β-lactamase; LOS: length of stay; ICU: intensive care unit

Outcome	ESBL+ (n=76)	ESBL- (n=96)	P-value
30-day mortality	23.70%	12.50%	0.06
90-day mortality	34.20%	17.70%	0.02
Median hospital LOS	15 days	10 days	0.004
ICU admission	44.70%	32.30%	0.1

Median hospital length of stay was longer in ESBL-positive cases (15 vs. 10 days, p=0.004), possibly reflecting delays in effective therapy and more complications. ICU admission was more frequent in ESBL-positive patients (33 cases, 44.7% vs. 31 cases, 32.3%), although this was not statistically significant (p=0.10).

Empirical carbapenem therapy was associated with a clinically substantial reduction in 30-day mortality compared with piperacillin-tazobactam (14.3% vs. 31.3%, p=0.12). This mortality benefit was more pronounced at 90 days (21.4% vs. 43.8%, p=0.07), suggesting sustained advantages of early administration of effective therapy. Delayed initiation of active antibiotics (>48 hours after blood culture collection) increased mortality risk nearly threefold (OR: 2.9, 95% CI: 1.5-5.6). Additionally, 68% of patients receiving inappropriate empirical therapy required ICU admission, compared with 32% of those started promptly on carbapenems (p=0.01). These findings support the use of carbapenems as first-line empirical therapy for suspected ESBL bacteremia in high-risk patients.

Among the 76 ESBL-producing infections identified, 35 cases (46.1%) were community-acquired. Urinary tract infection (UTI) was the most frequent source (22 cases, 62.9%), followed by pneumonia (six cases, 17.1%) and intra-abdominal infection (four cases, 11.4%). Skin and soft tissue infection (SSTI) and CLABSI were less common (two cases, 5.7% and 1 case, 2.9%), respectively (Table 3).

**Table 3:** Sources of infection in community-acquired ESBL-producing cases Distribution of infection sources among 35 community-acquired ESBL-producing Enterobacterales cases. UTI was predominant (22/35; 62.9%), followed by pneumonia (6/35; 17.1%) and intra-abdominal infection (4/35; 11.4%); SSTI (2/35; 5.7%) and CLABSI (1/35; 2.9%) were uncommon ESBL: extended-spectrum β-lactamase; UTI: urinary tract infection; SSTI: skin and soft tissue infection; CLABSI: central line-associated bloodstream infection

Source of infection	Number of cases	Percentage (%)
UTI	22	62.9
Pneumonia	6	17.1
Intra-abdominal infection	4	11.4
SSTI	2	5.7
CLABSI	1	2.9

UTIs were the predominant source, representing nearly two-thirds of community-acquired ESBL infections. Pneumonia and intra-abdominal infections were less frequent, while SSTI and CLABSI were rare. These findings emphasize the importance of considering UTIs as the primary source when managing suspected community-acquired ESBL infections, which may influence empirical antibiotic choices and guide outpatient infection control strategies.

**Figure 3:**
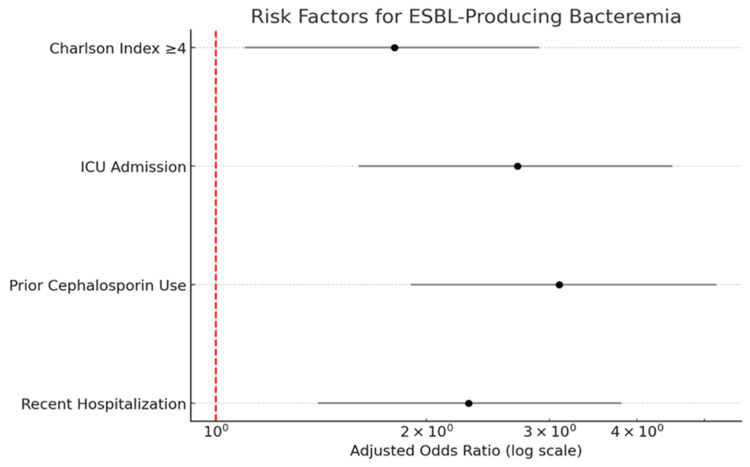
Forest plot of independent risk factors for ESBL-producing bacteremia Forest plot showing adjusted ORs (aORs) (dots) and 95% CIs (horizontal lines) for independent predictors of ESBL bacteremia. Prior cephalosporin use (aOR: 3.1, 95% CI: 1.9-5.2, p<0.001) was the strongest predictor, followed by ICU admission (aOR: 2.7, 95% CI: 1.6-4.5, p=0.002) and recent hospitalization (aOR: 2.3, 95% CI: 1.4-5.8, p=0.001). A CCI ≥4 was also associated (aOR: 1.8, 95% CI: 1.1-2.9, p=0.05). Model: multivariable logistic regression adjusted for age, CCI, Pitt Bacteremia Score, ICU admission, source of infection, and appropriateness of empirical therapy. ESBL: extended-spectrum β-lactamase; OR: odds ratio; CI: confidence interval; ICU: intensive care unit; CCI: Charlson Comorbidity Index

Multivariate logistic regression identified four independent risk factors for ESBL-producing bacteremia (Table 4, Figure 3). The strongest predictor was prior cephalosporin use (adjusted odds ratio (aOR): 3.1, 95% CI: 1.9-5.2, p<0.001), highlighting the impact of antibiotic selection pressure. ICU admission was also strongly associated with ESBL production (aOR: 2.7, 95% CI: 1.6-4.5, p=0.002), reflecting increased healthcare exposure and invasive device use. Recent hospitalization within 90 days was another significant risk factor (aOR: 2.3, 95% CI: 1.4-5.8, p=0.001), suggesting potential colonization from prior healthcare contact. A high comorbidity burden, defined as CCI >4, was also independently associated with ESBL bacteremia (aOR: 1.8, 95% CI: 1.1-2.9, p=0.05). The model demonstrated good discriminative ability with an area under the curve (AUC) of 0.78.

**Table 4:** Multivariate analysis of independent risk factors for ESBL-producing bacteremia Adjusted ORs with 95% CIs are presented from multivariate logistic regression. Prior cephalosporin use, ICU admission, and recent hospitalization were significant independent predictors of ESBL-producing bacteremia. A CCI ≥4 was also independently associated with increased risk. Model: multivariable logistic regression adjusted for age, CCI, Pitt Bacteremia Score, ICU admission, source of infection, and appropriateness of empirical therapy. Effect measure: adjusted OR (aOR) with 95% CI. Findings: prior cephalosporin use (aOR: 3.1; 95% CI: 1.9-5.2; p<0.001), ICU admission (aOR: 2.7; 95% CI: 1.6-4.5; p=0.002), recent hospitalization (aOR: 2.3; 95% CI: 1.4-5.8; p=0.001); CCI ≥4 (aOR: 1.8; 95% CI: 1.1–2.9; p=0.05). ESBL: extended-spectrum β-lactamase; OR: odds ratio; CI: confidence interval; ICU: intensive care unit; CCI: Charlson Comorbidity Index

Risk factor	Adjusted OR	95% CI	P-value
Recent hospitalization	2.3	1.4-5.8	0.001
Prior cephalosporin use	3.1	1.9-5.2	<0.001
ICU admission	2.7	1.6-4.5	0.002
CCI ≥4	1.8	1.1-2.9	0.05

## Discussion

Our study offers a detailed evaluation of BSIs caused by ESBL-E, yielding several key findings with important clinical implications. The prevalence of ESBL producers in our cohort was 43.8%, a figure that reflects the growing global burden of antimicrobial resistance, especially in healthcare settings with high antibiotic utilization [8]. This rate is higher than those reported in many Western countries but is consistent with findings from regions where ESBL prevalence is endemic [9].

The resistance patterns observed in this study highlight the significant therapeutic challenges posed by ESBL producers. High resistance rates to third-generation cephalosporins and fluoroquinolones restrict empirical treatment options, while the retained susceptibility to carbapenems reinforces their role as dependable therapeutic agents [10]. These findings are in line with published data from the UAE and the wider Gulf region, where ESBL-Es are highly prevalent, and carbapenems remain among the most reliable treatment options [11]. Our cohort, therefore, provides contemporary local susceptibility data that complement international guidance and can inform institutional antibiograms and stewardship policies. However, the emergence and spread of carbapenem-resistant Enterobacterales is well documented and highlights the need for antimicrobial stewardship [12].

In our retrospective analysis, patients with healthcare-associated bacteremia tended to be older and had higher CCI scores than those with community- or hospital-acquired infections. This pattern suggests increased vulnerability in these patients due to recent hospitalization, prior antibiotic exposure, or ongoing medical interventions [13]. The poorer clinical outcomes observed with piperacillin-tazobactam in ESBL bacteremia may be explained by the inoculum effect, in which apparent in vitro susceptibility does not translate to clinical efficacy when the bacterial burden is high [14,15]. This phenomenon suggests that conventional susceptibility testing may overestimate the real-world activity of piperacillin-tazobactam and cefepime against ESBL bacteremia. Current guidelines recommend avoiding these agents for ESBL BSIs, even if susceptibility is reported, and instead favor carbapenems for definitive therapy [10].

For critically ill patients or when *Pseudomonas aeruginosa* coverage is required, meropenem remains the preferred carbapenem. In stable patients without *Pseudomonas* risk, ertapenem offers a practical alternative and may facilitate outpatient parenteral antimicrobial therapy [10]. De-escalation to narrower-spectrum agents once susceptibility results are available supports stewardship without compromising patient outcomes [10]. Implementing structured antimicrobial stewardship strategies, including formal stewardship programs that have been shown to significantly reduce unnecessary antimicrobial use and costs, can further support early appropriate therapy while minimizing excessive carbapenem exposure [10,16]. In this context, risk-based use of empirical carbapenems in high-risk patients, followed by prompt de-escalation once susceptibilities are known, may help balance the need for timely, effective treatment with preservation of carbapenem activity.

One of the most clinically relevant observations of our study is the pattern of lower mortality among patients who received empirical carbapenem therapy. Patients who received empirical carbapenems had numerically lower 30-day mortality (14.3% vs. 31.3%, p=0.12) and 90-day mortality (21.4% vs. 43.8%, p=0.07) compared with those treated with piperacillin-tazobactam, although these differences did not reach statistical significance and should be interpreted cautiously in light of the observational design and potential confounding by indication. This real-world observation is directionally consistent with the MERINO randomized trial, which demonstrated the superiority of meropenem over piperacillin-tazobactam for ceftriaxone-resistant *E. coli* and* K. pneumoniae* bacteremia [17]. In our multivariable logistic regression model, delayed initiation of active antibiotics beyond 48 hours after blood culture collection was independently associated with nearly threefold higher odds of death (OR: 2.9, 95% CI: 1.5-5.6) [18], providing quantitative support for the link between timing of appropriate therapy and mortality.

Our risk factor analysis further identified patient populations most likely to benefit from empirical carbapenem therapy, including those with recent hospitalization, prior antibiotic use, or critical illness. These results are consistent with previous studies identifying recent healthcare contact, intensive care admission, and prior cephalosporin exposure as independent predictors of ESBL infection or bacteremia [19,20]. Targeted use of carbapenems in such high-risk patients can help balance the need for timely, effective therapy with the imperative to minimize unnecessary broad-spectrum antibiotic exposure [10].

Limitations

This study has several limitations. The retrospective design introduces potential for confounding, though we attempted to control for this through multivariable analysis. In particular, confounding by indication cannot be excluded in the comparison of empirical carbapenem versus non-carbapenem regimens, because sicker patients may have been more likely to receive carbapenems. Our single-region experience may limit generalizability, particularly to settings with different resistance patterns. The one-year study period and modest sample size may also limit the precision of some estimates, and larger multicenter cohorts would help to confirm and refine these findings. In addition, carbapenem-resistant isolates were excluded, so the results do not address treatment strategies for carbapenem-resistant *Enterobacterales*. Finally, we were unable to evaluate newer β-lactam/β-lactamase inhibitor combinations that may offer carbapenem-sparing alternatives.

## Conclusions

This study demonstrates that ESBL-E BSIs are associated with significant morbidity and mortality, particularly when appropriate therapy is delayed. Delayed initiation of active therapy beyond 48 hours was independently associated with nearly threefold higher odds of death (OR: 2.9, 95% CI: 1.5-5.6), underscoring the importance of early appropriate treatment. After excluding carbapenem-resistant cases, our findings show complete susceptibility to carbapenems in ESBL producers (0% resistance to ertapenem and meropenem) and are consistent with a possible survival advantage of empirical carbapenem therapy in high-risk patients, although mortality differences compared with piperacillin-tazobactam did not reach statistical significance and should be interpreted as hypothesis-generating rather than definitive. The results highlight the need for rapid diagnostic methods to facilitate early targeted therapy. Antimicrobial stewardship programs should focus on optimizing empirical carbapenem use in high-risk populations while preserving these critical agents through de-escalation when possible. Future research should evaluate the role of newer β-lactam/β-lactamase inhibitors and rapid diagnostic technologies in improving outcomes for these challenging infections. At a health-system level, integrating these local susceptibility and outcome data into national antimicrobial resistance surveillance efforts in the UAE may help refine empiric therapy guidelines and stewardship priorities.
